# A plasma mir-125a-5p as a novel biomarker for Kawasaki disease and induces apoptosis in HUVECs

**DOI:** 10.1371/journal.pone.0175407

**Published:** 2017-05-03

**Authors:** Zhuoying Li, Jie Jiang, Lang Tian, Xin Li, Jia Chen, Shentang Li, Chunyun Li, Zuocheng Yang

**Affiliations:** Department of Pediatrics, The Third Xiangya Hosptial of Central South University, Changsha, Hunan, P.R.China; Saint Louis University, UNITED STATES

## Abstract

**Background:**

Kawasaki disease (KD) is a childhood systemic vasculitis that exhibits a specific preference for the coronary arteries. The aetiology remains unknown and there are no especially diagnostic tests. microRNAs (miRNAs) are 18 to 23 nucleotides non-coding RNAs that are negative regulator of gene expression and play a crucial role in the regulatory network of the genome. Recently, circulating miRNAs have been found presentation in human plasma and displayed some characteristics of the ideal biomarker. However, few researches explored differentially expressed miRNAs in the plasma of KD patients. Our study is to identify circulating miRNAs in KD plasma which can serve as potential biomarkers of KD diagnosis.

**Materials and methods:**

The total of five pairs of acute KD and normal plasma samples were analyzed using ABI miRNAs TLDA Assay chip. Differentially expression of miR-125a-5p in plasma were confirmed by quantitative real-time PCR (qRT-PCR) in independent cohort (acute KD = 30, convalescent KD = 30 and healthy control = 32). After bioinformatics prediction, miR-125a-5p vector and inhibitor were transfected into HUVECs respectively, to observe MKK7 expression as a potential target gene. Flow cytometry was used to analyze apoptosis. The mRNA and protein levels of desired genes including MKK7, Caspase-3, Bax and Bcl2 were detected by qRT-PCR and western blotting.

**Results:**

Eighteen miRNAs were differentially expressed in acute KD’s plasma compared with healthy control. miR-125a-5p was significantly increased in plasma of KD patients (*p* = 0.000), but no variation between acute and convalescent KD (*p* = 0.357). Moreover, the results from the gain and loss functions of miR-125a-5p in HUVECs have shown that miR-125a-5p remarkably suppressed MKK7 expression, as a novel target gene. Importantly, miR-125a-5p also induced apoptosis in HUVECs through inhibition MKK7 levels to regulate Bax/Bcl2 pathway resulting to activate Caspase-3.

**Conclusion:**

Our study indicated that the circulating miR-125a-5p levels in KD’s plasma have remarkably evaluated compared with healthy individuals. miR-125a-5p might play a role in the development of KD by regulating target gene MKK7 to induce apoptosis in vascular endothelial cells. Therefore, our findings have suggested that detected miR-125a-5p levels in plasma could be used as a potential biomarker in early KD diagnosis.

## Introduction

Kawasaki disease (KD) is a self-limited childhood systemic vasculitis that is characterized by a specific preference for the coronary arteries. KD results in coronary arteries abnormalities is up to 25% of children if untreated [1]. The current view is that genetically susceptible children are exposed to an unknown trigger that elicits an immune response directed against some components of the arterial wall [2]. However, the exact mechanisms of KD have yet known. So far, the diagnosis of KD is based on the clinical features, and a specific laboratory test for the early identification and diagnosis of KD does not exist [3]. Importantly, delay in accurate diagnosis comes out increased mortality and morbidity from complications of KD [4]. microRNAs (miRNAs) are 18 to 23 nucleotides non-coding RNAs. miRNAs mediated mRNA cleavage, translational repression or mRNA destabilization [5]. The amounts of research reports have demonstrated that miRNAs are involved in various biological processes, such as cellular proliferation, apoptosis, angiogenesis and differentiation [6–8]. Recently, the nuclease-resistant extracellular miRNAs have been found that they are present in human blood plasma [9, 10], and displayed some characteristics of an ideal biomarker. There are a mounting number of reports identifying specific plasma miRNAs as potential biomarkers including cancer [11], heart failure [12], myocardial infarction [13] and so on. However, few studies have explored plasma miRNAs expression difference in KD patients.

In current study, we first profiled differentially expressed miRNAs in the plasma of acute KD patients and healthy controls. According to the results of miRNAs assay chip, we have identified a miRNAs panel including 11 miRNAs upregulated and 7 miRNAs downregulated in the plasma of KD patients compared with control group. Based on these different plasma miRNAs might play a role in regulating gene expression, we have focused on one of differentially expressed miRNAs, miR-125a-5p, due to its role in impaired endothelial cell angiogenesis [14], and we have validated that miR-125a-5p was remarkably upregulated in KD patients’ plasma in comparison with controls. In addition, we also found that augmentation or repression of miR-125a-5p in human umbilical vein endothelial cells (HUVECs) leaded to induction or inhibition apoptosis, and miR-125a-5p induced apoptosis in HUVECs, at least, partially dependent on inhibiting the novel target gene MKK7.

## Materials and methods

### Clinical information and blood samples collecting

From January 2010 to December 2013, consistent with Kawasaki Disease diagnostic criteria, the children were confirmed by two or more pediatricians. The follow-up of all patients were record clinical and laboratory information including blood routine, C-reactive protein (CRP), erythrocyte sedimentation rate (ESR), cardiac enzyme level, liver and renal function, whether there is any abnormal coronary artery by cardiac color ultrasound. At same time, the children by diagnosed KD were given intravenous γ-globulin (IVIG) and oral aspirin and dipyridamole treatment, the control samples were collected according of gender and age matching. Whole blood (2 ml) before treatment was drawn into EDTA-containing tubes. Blood samples were centrifuged to spin down the blood cells, and the supernatant was transferred into microcentrifuge tubes, followed by second centrifugation completely remove cellular components. Plasma was then aliquoted and stored at -80°C until used. This study has been approved by ethics committee of the Third Xiangya hospital, central south university. Informed consents of patients and healthy controls were obtained from each parents by writing official paper.

### Profiling plasma miRNAs and data analysis

To plasma miRNAs profiling assay, the total of five pairs of acute KD and normal plasma samples were randomly selected. Total RNAs containing small RNAs were extracted by using Trizol LS reagent (Invitrogen) and mirVanaTM miRNA Isolation Kit (Ambion) according to the manufacturer’s protocol. The concentration of all RNA samples were quantified, then three microliters of total RNA were reverse transcribed using TaqMan MicroRNA Reverse Transcription Kit (Applied Biosystem) to synthesis miRNA specific cDNA, the cDNA were pre-amplified for 12 cycles by using the corresponding MegaplexTM RT primers Human pool A v2.1 and Human pool B v3.0 (Applied Biosystem). The pre-amplified cDNAs were performed miRNA expression profiling by using TaqMan Human MicroRNA Assay A (Applied Biosystem) containing 768 mature human miRNAs according to the commercial protocol. The signal detection was performed by 7900HT fast RT-PCR System (Applied Biosystem).

To analysis miRNAs expression, miRNAs were first normalized using global mean normalization across all miRNA TaqMan arrays to achieve the same mean Ct, and miRNAs with Ct>35 in all groups were filtered out for minimization potential noise. Differentially expressed miRNAs were confirmed by volcano plot. Cluster analysis was performed by using Partek Genomic Suite (Partek Inc.). The threshold value for candidate differentially expressed miRNAs was a fold change >2.0 with a value of *p*<0.05.

### Differential miRNAs confirmation and genes mRNA expression by quantitative real-time PCR

To confirm the results from miRNAs profiling assay, the quantitative real-time PCR was used to evaluate the expression levels of miR-125a-5p. Total RNAs were extracted from the plasma of Kawasaki disease and healthy controls. In brief, 40ng plasma RNA containing miRNA was polyadenylated bypoly(A) polymerase and reverse transcribed into cDNA using miScript Reverse Transcription Kit (Qiagen). Real-time PCR was performed miScript SYBR Green PCR Kit (Qiagen) with the human miR-125a-5p specific forward primer in ABI 7900HT Real-time PCR system (Applied Biosystems). Because we could not detect U6 or 5S in plasma samples, based on previously publications [15] and as recommended by the manufacturer (Applied Biosystems), we used the levels of miR-16 in plasma for normalization. Then, the expression levels of miR-125a-5p were normalized to miR-16. The miR-125a-5p and miR-16 specific primers sequences were

MiR-125a-5p: 5’-TCCCTGAGACCCTAACTTGTGA-3;
MiR-16: 5'-TAGCAGCACGTAAATATTGGCG-3'

For genes mRNA expression detection, total RNAs were extracted from the cultured cells using Trizol reagent (Invitrogen) and reverse transcription reactions were performed to obtain the first cDNA strand, then and quantitative real-time PCR was performed in triplicate with ABI 7900HT Real-time PCR system using SYBGreen PCR Master Mix (Roche). Relative mRNA and miRNA expression expression level were normalized to Actin or miR-16 and relative expression fold change was calculated with the 2^-CT^ method. Genes’ primers sequences are provided in S1 Table.

### Cell culture and transfection

The human umbilical vein endothelial cell lines (HUVECs) were cultured in DMEM with high glucose supplemented with 10% FBS and 1% penicillin-streptomycin. All cells were cultured in a humidified atmosphere of 95% air and 5% CO2 at 37°C. The recombinant plasmids (GV230-GFP) were constructed by amplifying miR-125a-5p precursors from a library of vectors expressing miRNAs and then connecting it. miR-125a-5p inhibitors were purchase from Qiagen. The transfection with miR-125a-5p vector or inhibitor was performed using Lipofectamine2000^TM^ reagent. The expression of miR-125a-5p was tested by Real-time PCR and fluorescence microscope.

### Luciferase assay

Human 3’-UTR of MKK7 was cloned into a luciferase reporter (pLUC-MKK7, Promega, Madison MA, USA) by inserting a 592-bp 3’-UTR DNA fragment spanning the predicted miR-125a-5p target site. An empty luciferase reporter plasmid (pLUC-Control) was used as the control. HUVECs were transiently co-transfected with miR-125a-5p and 5μg luciferase reporter vectors using Lipofectamine2000^TM^ reagent for 48 hours. The relative luciferase activities were evaluated by a dual-luciferase reporter assay (Promega, Madison MA, USA) according to the manufacturer’s protocol.

### Western blotting

Total proteins were obtained by a RIPA lysis buffer (Santa Cruz). Protein samples were loading in 10% SDS-PAGE gel and transferred to polyvinylidene fluoride (PVDF) membranes (Millipore). The membranes were incubated with primary antibodies including MKK7 (ProteinTech), Bcl-2 (Bioss, Beijing, China), Bax (Bioss, Beijing, China) and caspase-3 (Cell signaling Technology). The blots were incubated with enhanced chemiluminescence substrate (Bio-Rad), and then visualized.

### Apoptosis by flow cytometry

To observe apoptosis for transfected miR-125a-5p overexpression or inhibitor in HUVECs, we have performed the flow cytometry experiment. In brief, HUVECs were harvested at the indicated time points. After double staining with FITC-Annexin V and propidium iodide (Beyotime, China), the cells were analyzed with a flow cytometry FACS Carton II equipped with Diva software (BD Bioscience). Measurements were repeated independently three times.

### Statistical analysis

Plasma miRNA levels were not normally distributed, so the significance of plasma miRNA levels was determined by the Mann-Whitney test. Box-plots were performed with a log^10^ transformation of data. SPSS 16.0 software was used for statistical analysis. Data were presented as mean±SD. Two-tailed Student's t test was used for comparisons of two independent groups. Three or more independent groups were analyzed by One-way ANOVA. By using the LSD tests for multiple comparisons. P value of less than 0.05 was considered statistically significant.

## Results

### Acute KD plasma miRNA profiling assay

**Fig 1:**
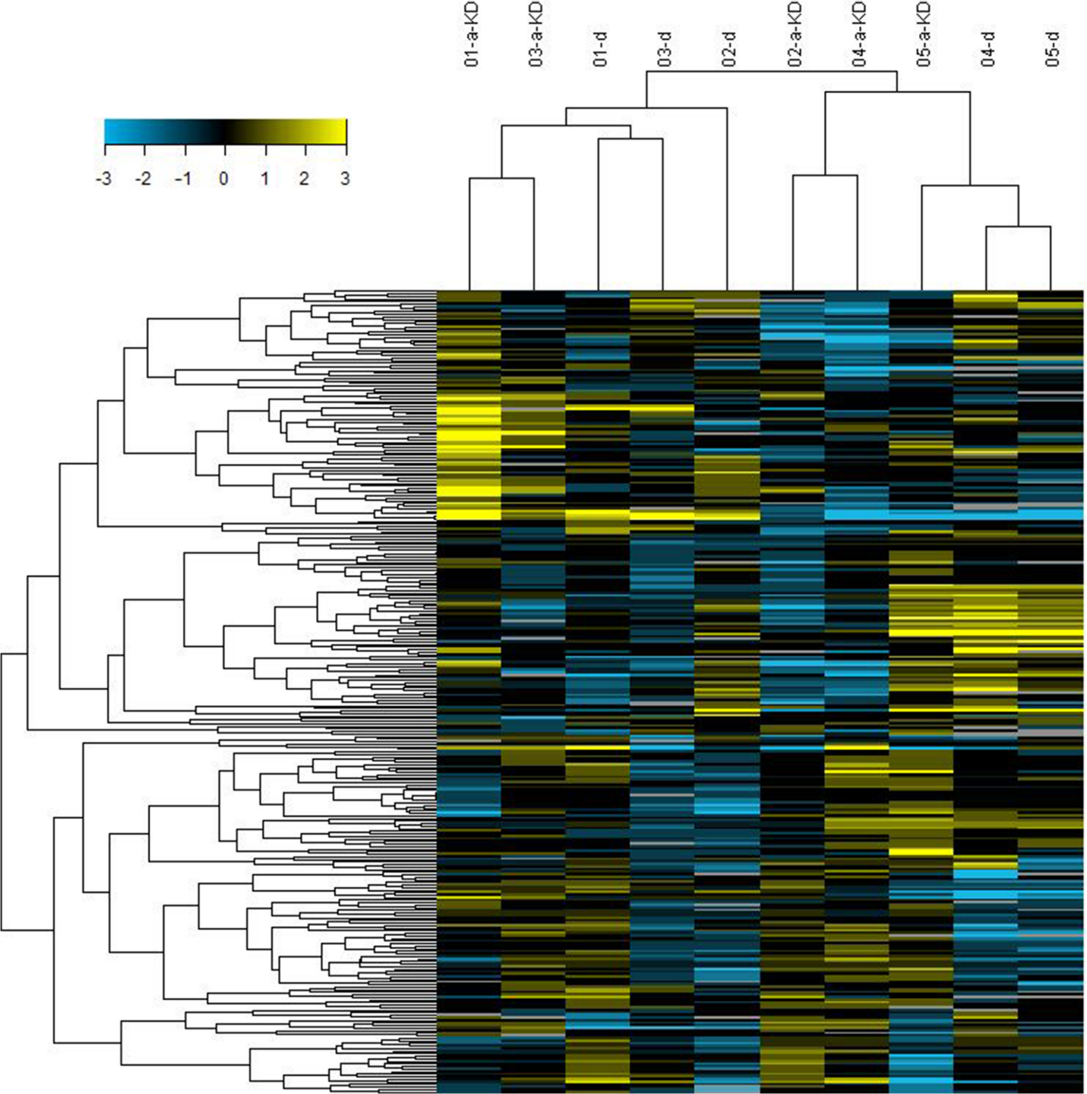
Cluster analysis of the plasma miRNAs expression in five pairs of acute Kawasaki disease patients and healthy control samples. A-KD refers to acute Kawasaki Disease; d refers to healthy control sample. Lower/higher miRNAs relative to the mean of all samples were shown as blue/yellow.

We tested five pairs of plasma from acute KD patients and healthy controls. Through the 768 candidates analyzed using the AB miRNA TLDA array approach, 18 miRNAs were differentially expressed in acute KD’s plasma compared to healthy control’s plasma (Fig 1). The expression levels of 11 plasma miRNAs were more than two-fold higher in acute KD patients, and 7 plasma miRNAs of acute KD patients have decreased expression, the decreased range of the expression fold from 0.1909 to 0.4729 (Table 1).

**Table 1:** Differential expression of the plasma miRNAs in acute KD compared to healthy control samples.

miRNA Assay	Relative Fold of Expression	*P* Value
**Up-regulation** hsa-miR-125a-5p	2.7534	0.0487
hsa-miR-133a	2.274	0.0402
hsa-miR-148a	2.2816	0.0191
hsa-miR-199b-5p	2.493	0.0287
hsa-miR-223	2.3156	0.0288
hsa-miR-330-3p	3.2977	0.0125
hsa-miR-483-5p	2.2335	0.0308
hsa-miR-671-3p	2.5286	0.0234
hsa-miR-744	2.2899	0.0254
hsa-miR-885-5p	5.009	0.0149
hsa-miR-7	2.1275	0.0400
**Down-regulation** hsa-miR-10b	0.3349	0.0386
hsa-miR-138	0.1909	0.0239
hsa-miR-29b	0.3908	0.0040
hsa-miR-455-3p	0.346	0.0428
hsa-miR-455-5p	0.2617	0.0127
hsa-miR-516-3p	0.4729	0.0304
hsa-miR-627	0.2548	0.0255

### Confirm the plasma miR-125a-5p upregulation in acute KD

**Fig 2:**
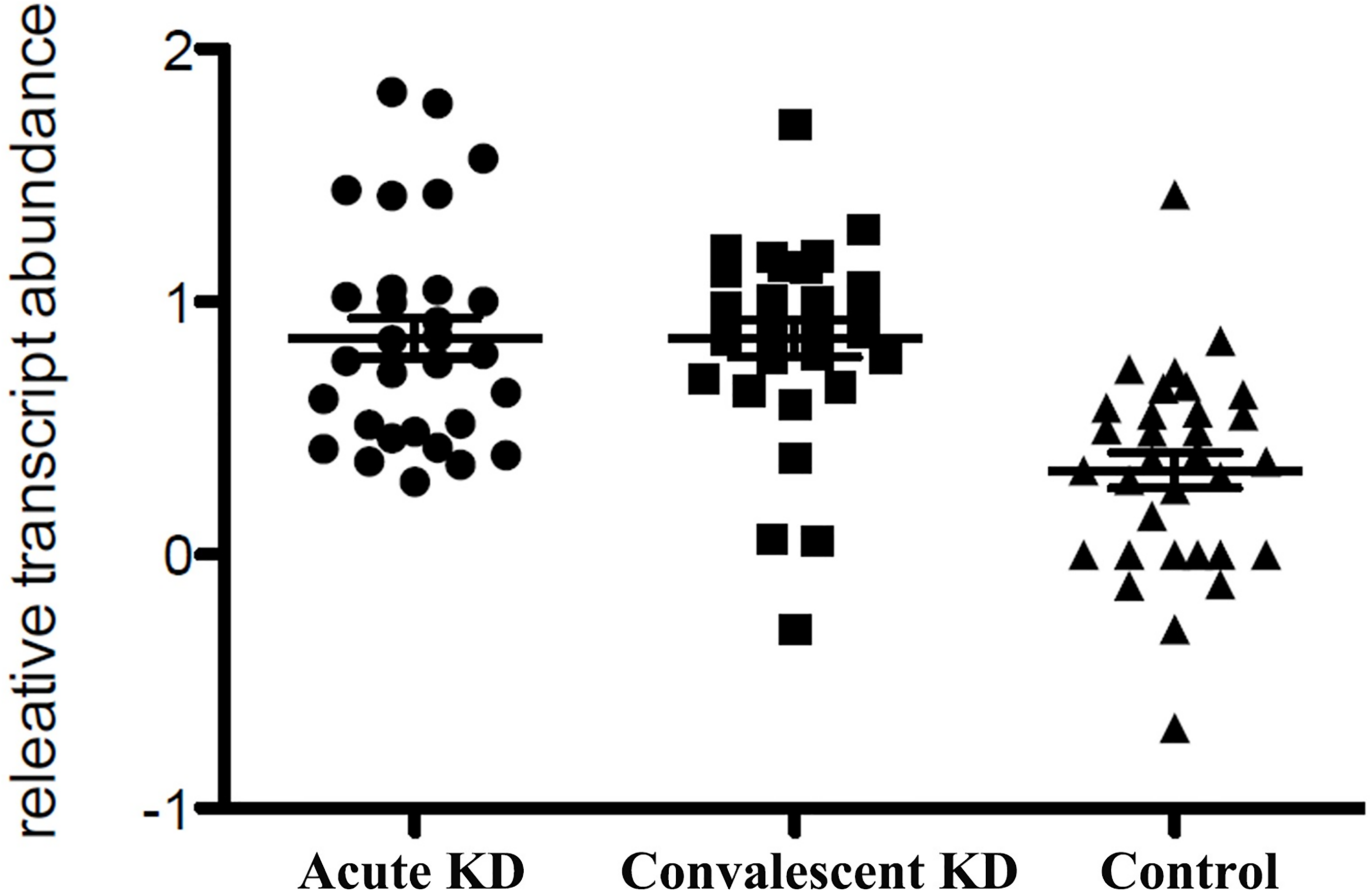
Scatter diagram of the plasma miR-125a-5p levels in acute, convalescent KD patients and healthy control samples. Acute KD = 30 samples; Convalescent KD = 30 samples; control = 32 samples.

Based on the ratio of expression and previously reports, we selected miR-125a-5p as candidate maker, which increased 2.75-fold in acute KD patients plasma compared to healthy control samples, to confirm by employing real-time PCR. We performed real-time PCR in independent cohort plasma including 30 acute KD patients, 30 convalescent KD patient and 32 healthy control samples respectively. Due to miR-16, which was consistent among our all samples, and previously used as the internal control for plasma miRNAs analysis [16], we used miR-16 levels for data normalization. Consistent with our finding in miRNA profiling assay, the plasma miR-125a-5p levels were significantly higher in both of acute and convalescent KD patients than healthy control samples (Table 2), however, there was no statistic difference between acute and convalescent KD patients (*p* = 0.357) (Fig 2). Taken together, the expression levels of miR-125a-5p were remarkably altered in KD patients plasma compared with the healthy controls.

**Table 2:** The plasma expression levels of miR-125a-5p in acute, convalescent KD patients and healthy control samples.

Groups	Numbers	2^-△△Ct^	*χ*^2^	*P*
Control	32	2.58±1.66	32.66	0.000
Acute KD	30	12.63±16.51		
Convalescent KD	30	9.92±8.98		

The difference of 2^-△△Ct^ in three groups was analyzed with Kruskal-Wallis H, there were significant difference (*p*<0.05). The 2^-△△Ct^ data of each groups were transformed to rank and pairwise comparison by LSD analysis. The results have shown that the plasma miR-125a-5p levels were significantly increased in acute and convalescent KD patients p = 0.000, p = 0.000) compared to control, but no difference between acute and recovery KD patients (p = 0.357). KD: Kawasaki Disease.

### MKK7 is a novel target gene of miR-125a-5p in HUVECs

**Fig 3:**
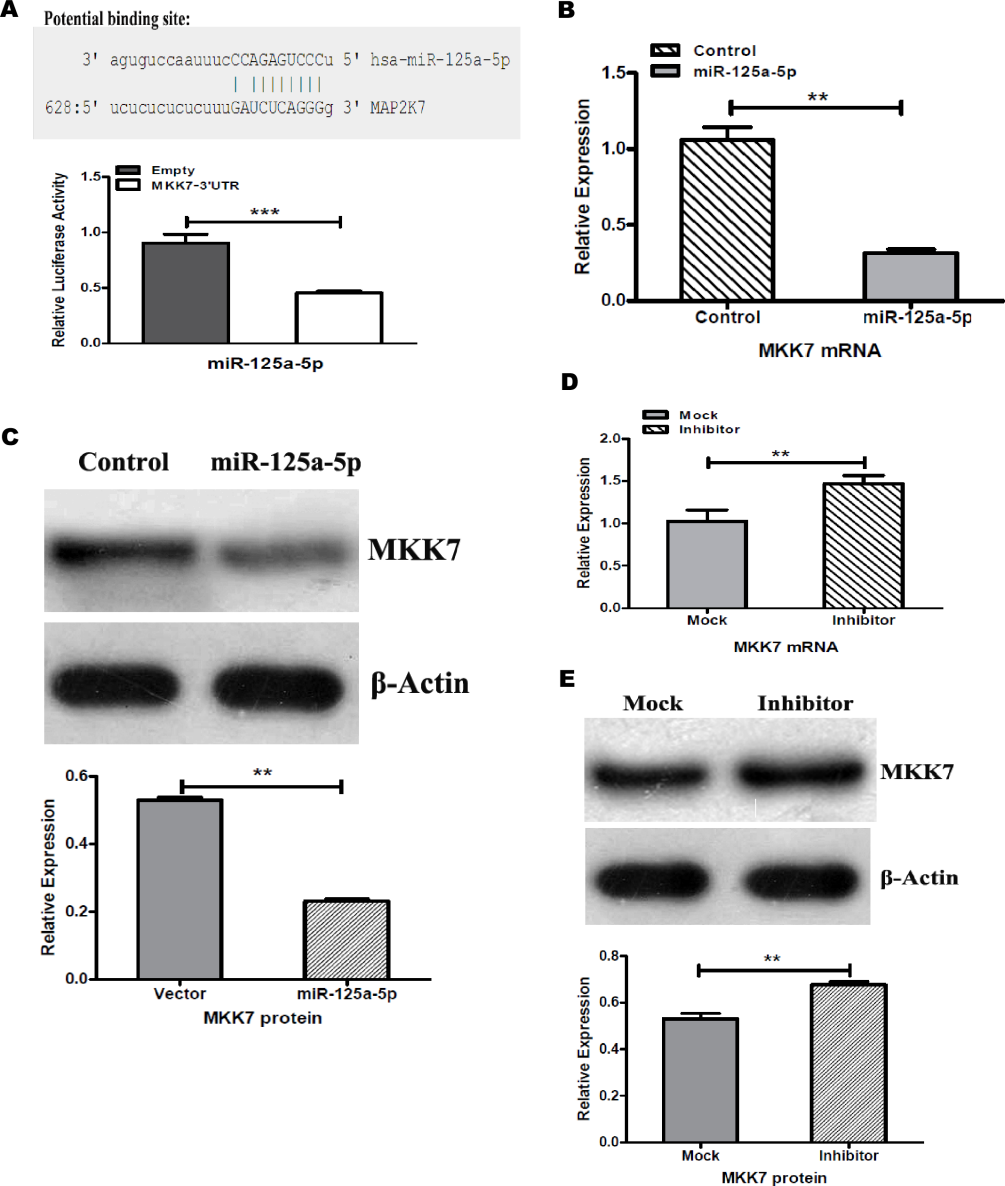
MKK7 was a novel target gene for miR-125a-5p. (A) Putative miR-125a-5p binding site predicted by miRanda database in 3’-UTR of human MKK7 gene (MAP2K7) (up). Transient luciferase reporter assays. Constructed the fragment of MKK7 3’-UTR or empty luciferase reporter vectors were transfected into HUVECs together with miR-125a-5p-mimic. Co-transfection after 48hr, a dual-luciferase reporter assay was performed. The luciferase activities were measured for all vectors and then normalized to the luciferase activity of Renila vector (***p<0.01) (down). Transfection of miR-125a-5p overexpression vector significantly reduced MKK7 mRNA (B) and protein (C) levels in HUVECs as compared with control vector. Using miR-125a-5p inhibitor remarkably enhanced mRNA and protein expression of MKK7 in HUVECs as compared with mock transfection (D and E). All results are representative of three independent experiments. **p<0.05

First, we predicted the target genes of miR-125a-5p by using five public databases including miRanda, MirTarget, miCroT_V3.0, PicTar and TargetScans. The same target genes predicted by more than three databases algorithms were selected as candidate targets, then we performed GO enrichment analysis in these candidate targets, we found that there are one putative miR-125a-5p binding site in the 3’-UTR of the MKK7 (MAP kinase kinase 7) mRNA (Fig 3A, up). Importantly, we constructed the 3’-UTR region of the MKK7 including a miR-125a-5p target site vector, and the luciferase reporter assay indicated that the 3’-UTR region of the MKK7 is responsible for the miR-125a-5p mediated inhibition in human umbilical vein endothelial cells (Fig 3A, down). Then, we have identified that MKK7 was one of the novel target genes of miR-125a-5p in HUVECs.

To further verify MKK7 as a novel target, we detected the mRNA and protein levels of MKK7 while transfection with miR-125a-5p expression vector or inhibitor in HUVECs (S1 Fig). The results have shown that miR-125a-5p overexpression dramatically inhibited mRNA and protein expression of MKK7 compared with control transfection (Fig 3B and 3C). In addition, we decreased miR-125a-5p expression in HUVECs using anti-miR-125a-5p inhibitor. As expected, the expression of MKK7 including mRNA and protein levels were significantly increased (Fig 3D and 3E). Together, these results established that MKK7 expression is regulated by miR-125a-5p, and MKK7 is a novel target gene of miR-125a-5p in HUVECs.

### MiR-125a-5p induces apoptosis via inhibiting MKK7 expression in HUVECs

**Fig 4:**
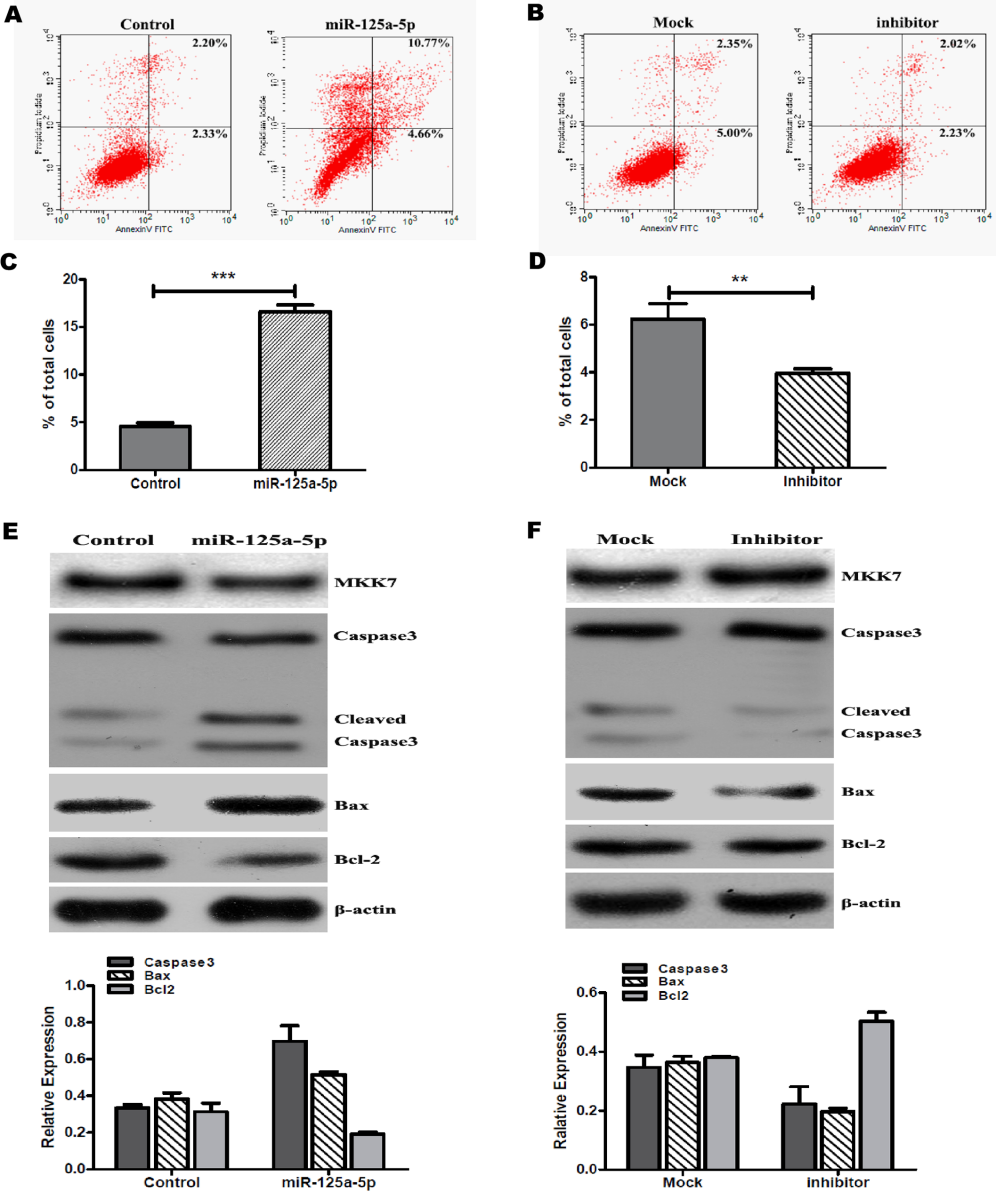
miR-125a-5p induces apoptosis in HUVECs. Representative dot plots of staining with Annexin V and PI. After HUVECs were tranfected with miR-125a-5p expression vector (A) or inhibitor (B) for 48hr, then performed apoptosis analysis by flow cytometry. (C) Comparison of apoptotic cells in miR-125a-5p overexpression and control. The percentage of apoptosis cells was significantly higher in increased miR-125a-5p expression. (D) Inhibition of miR-125a-5p in HUVECs reduced the percentage of apoptosis. The expression of apoptotic proteins detected when HUVECs were tranfected with miR-125a-5p expression vector (E) or inhibitor (F) for 48hr. The data are presented as mean±SD. All results are representative of three independent experiments. **p<0.05, ***p<0.01.

As we known, MKK7 is involved in the regulation of JNK (c-Jun N-terminal kinase) signaling pathway, and JNK signaling pathway affects a variety of intracellular events including cell apoptosis [17]. To determine whether miR-125a-5p induced apoptosis by affecting MKK7 pathway in HUVECs. First, we observed cell apoptosis by staining with Annexin V and PI after transfection miR-125a-5p expression vector or inhibitor in HUVECs. Flow cytometry analysis has showed that increased miR-125a-5p profoundly triggered HUVECs apoptosis (Fig 4A and 4C). Inversely, decreased miR-125a-5p expression remarkably inhibited HUVECs apoptosis (Fig 4B and 4D).

At same time, we detected the expression of apoptotic proteins such as Bcl2, Bax and caspase-3 after HUVECs were transfected with miR-125a-5p vector or inhibitor. The results have shown that overexpression of miR-125a-5p significantly reduced MKK7 expression leading to increased the activities of caspase-3, induced pro-apoptotic protein Bax expression and inhibited the levels of anti-apoptotic Bcl-2 expression (Fig 4E). Consistently, inhibition miR-125a-5p in HUVECs increased MKK7 levels resulting in reducing caspase-3 activation, decreasing Bax expression and increasing anti-apoptotic Bcl-2 levels (Fig 4F). Taken together, miR-125a-5p induces apoptosis via inhibiting MKK7 expression in HUVECs.

## Discussion

In current study, we firstly identified 18 differentially expressed miRNAs by using the miRNA TLDA array chip approach in the plasma of acute KD and healty control. Among these, miR-125a-5p, 133a, 148a, 199b-5p, 223, 330-3p, 483-5p, 671-3p, 744, 885-5p and miR-7 were significantly increased in acute KD plasma compared with control group, however, the expression levels including miR-10b, 138, 29b, 455-3p, 455-5p, 516-3p and miR-627 were downregulated in the plasma of acute KD in contrast to healthy control. Recently, circulating miRNAs in biological fluids, especially in human plasma or serum, have emerged as potential biomarkers for the detection of various diseases [18–20]. Despite high RNase concentrations in human blood, circulating miRNAs were found to be extremely stable, even underwent extreme variations in pH, multiple freeze-thaw cycles and extended storage [21, 22]. To date, there are no specific tests and biomarkers to diagnose Kawasaki disease (KD) in early, based on the characteristics of circulating miRNAs, we attempted to find new biomarkers from plasma miRNAs for early diagnosis of KD. In the previous research, Chisato et al have studied miRNAs from whole blood during the acute and convalescent KD patients, and revealed that six miRNAs were significantly elevated during the acute phase of KD, miR-145, one of the differential miRNAs, might involved in the progression of acute KD dependent on modulating TGF-β signaling pathway [23]. Our study focused on the differential plasma miRNAs expression profile between acute KD and healthy control group, and found that the plasma miR-125a-5p levels were significantly higher in both of acute and convalescent KD patients than healthy control, however, there was no statistic difference between acute and convalescent KD patients. The current finding has suggested that miR-125a-5p has an important role in the pathophysiology of KD, and detection of plasma miR-125a-5p levels might be used as a new biomarker for early KD diagnosis.

Recently reports, miR-125a-5p plays an important role in angiogenesis via suppressing endothelin-1 (ET-1) expression in human umbilical endothelial cells (HUVECs) [24]. In aging mice, miR-125a-5p has an anti-angiogenic effect by indirectly regulating VEGF and eNOS expression [14]. Interestingly, miR-125a-5p was shown to reduce the expression of VEGF and MMP11 in hepatocellular carcinoma cells [25]. According to these proof, we hypothesized that miR-125a-5p is associated with the functions of blood vessel in KD. Then, we predicted the potential targets genes of miR-125a-5p using five public miRNAs databases, and identified MKK7 as a potential target. Consistently, as the dual-luciferase report assay have already determined that miR-125a-5p directly bonded the 3’-UTR region of MKK7 (Fig 3A), we also demonstrated that MKK7 was remarkably inhibited in HUVECs while miR-125a-5p was overexpressed (Fig 3B and 3C). Conversely, decreased miR-125a-5p in HUVECs induced the expression levels of MKK7 (Fig 3D and 3E). Therefore, our results indicated that miR-125a-5p regulated the novel target MKK7 expression and affected the functions in HUVECs.

MKK7 is a mitogen-activated protein kinase kinase that is activated in response to various cellular stresses [26]. Especially, MKK7 plays an important role in pro-apoptotic effects in various cell types [27, 28]. Generally, MKK7, as a part of JNK signaling pathway, transduces pro-apoptotic signals and inhibition of MKK7 contributes to apoptosis suppression [29–31]. However, also some studies have shown that MKK7 downregulation involved in apoptosis induction and cell cycle resting. For example, histone deaceyltrasferases inhibitors (HDACIs) downregulated MKK7 expression resulting in JNK/c-Jun pathway inactivation and inhibition proliferation in neuroblastoma cell lines [32]. And the researchers also have shown that blockade of the NF-κB ability to shut down MKK7 may promote apoptosis of self-reactive/pro-inflammarory cell and, perhaps, of cancer cells [33]. So, we have deduced that miR-125a-5p involved in HUVECs apoptosis by regulating MKK7. To further explore the functional effect of miR-125a-5p and MKK7, we have overexpressed miR-125a-5p or inhibited endogenous miR-125a-5p in HUVECs, consistent with our expectations, increased miR-125a-5p profoundly triggered HUVECs apoptosis (Fig 4A and 4C). Inversely, decreased miR-125a-5p expression suppressed HUVECs apoptosis (Fig 4B and 4D). Take into account our finding, we have concluded that miR-125a-5p induces apoptosis via inhibiting MKK7 expression in HUVECs. Certainly, as we known, there are the functionally redundant between MKK7 and MKK4. MKK7 and MKK4 are both activate the JNK pathway [34]. Then, we have speculated that MKK7 inhibition via miR-125a-5p would lead to MKK4 altered to execute the functionally redundant for JNK pathway activation resulting in apoptosis induction in HUVECs. Yet, this hypothesis might be more explored in the future.

Furthermore, the circulating miRNAs have been verified and applied in cancers diagnosis and prognosis and many other diseases [35–37]. The researches in the circulating miRNAs functions have been put on processing. However, the possible functions and mechanisms of the circulating miRNAs remain unclear. So far, some of researches have revealed that the plasma circulating miRNAs have derived of apoptosis or necrosis cells, or active release from cells and circulating cytolysis, and have triggered the functional effects [38]. Other studies have shown that the circulating miRNAs can be secreted via cell-derived microvesicles including microparticles and exosomes, and can transfer the gene-silencing signal between living cells *in vitro* and *in vivo* [39]. Recently report has demonstrated that ex-miRNAs can be shielded from RNase degredation by packaging them into membrane vesicles (MVs) including apoptotic bodies, microvesicles and exosomes or by complexing with AGO protein [40]. However, some investigators found that, at least in blood plasma and in media collected from common human cell lines, 95–99% of ex-miRNAs are vesicle-free and instead travel within AGO protein-positive ribonucleoprotein (RNP) particles [22, 41]. In current study, we did not detect microvesicles in plasma samples of KD, whether the plasma miR-125a-5p derived from cell secretion, this has yet known. On the other hand, according to miR-125a-5p induced HUVECs apoptosis, we have supposed that the plasma miR-125a-5p has increased in acute and convalescent KD patients, one reason might be released cellular miR-125a-5p from apoptosis or necrosis vascular endothelial cells. However, further studies regarding these issues are required in our future research.

In conclusion, using a comprehensive array method to analyze plasma miRNAs from KD patients, we have identified 11 miRNAs upregulated and 7 miRNAs downregulated in the plasma of KD patients compared with healthy controls. Subsequently, we have demonstrated that the plasma miR-125a-5p levels were significantly higher in both of acute and convalescent KD patients than healthy control samples. Our finding has suggested that miR-125a-5p may be potential diagnostic biomarkers for early KD. Importantly, a role for miR-125a-5p in HUVECs apoptosis regulation via a novel target gene MKK7 was elucidated for the first *in vitro*. Certainly, the mechanisms and sources of extra-cellular miR-125a-5p will also clarify the role of miR-125a-5p as biomarkers in early diagnosis of KD. Eventually, we have provided clues for further study of the relationship between miRNAs and KD development, and to provide molecular markers in order to improve the diagnosis early for KD ultimately.

## Supporting information

S1 Table Genes’ primers sequences for this study. (DOCX)

S1 Fig Transfection with miR-125a-5p expression vector or inhibitor in HUVECs. (DOC)
