# Supplementary material for: A plasma mir-125a-5p as a novel biomarker for Kawasaki disease and induces apoptosis in HUVECs
Source: PLoS One. 2017 May 3;12(5):e0175407. doi: 10.1371/journal.pone.0175407 (PMC5415180; doi:10.1371/journal.pone.0175407)
Supplement: S1 Table — (DOCX) [file pone.0175407.s001.docx]

**S1 Table 1**: **Genes’ primers sequences for this study**

| **Gene** | **Primers** | **Product size** |
| --- | --- | --- |
| MKK7 | F: 5’- GATGACAGTGGCGATTGTGAAGGC-3’ | 100bp |
|  | R: 5’-CCGCTCGTCCAGCAGGATGTT-3’ |  |
| Caspase-3 | F: 5’-TGTGAGGCGGTTGTAGAAGAGT-3’ | 157bp |
|  | R: 5’-CACACCCACCGAAAACCAGAG-3’ |  |
| Bcl-2 | F: 5’-CCTATCTGGGCCACAAGTGAA-3’ | 122bp |
|  | R: 5’-ACAGCCTGCAGCTTTGTTTC-3’ |  |
| Bax | F: 5’-GCCCTTTTGCTTCAGGGTTTC-3’ | 211bp |
|  | R: 5’-GGAAAAAGACCTCTCGGGGG-3’ |  |
| β-actin | F: 5’- CATCCTGCGTCTGGACCTGG-3’ | 107bp |
|  | R: 5’-TAATGTCACGCACGATTTCC-3’ |  |
