# Supplementary material for: A plasma mir-125a-5p as a novel biomarker for Kawasaki disease and induces apoptosis in HUVECs
Source: PLoS One. 2017 May 3;12(5):e0175407. doi: 10.1371/journal.pone.0175407 (PMC5415180; doi:10.1371/journal.pone.0175407)
Supplement: S1 Fig — (DOC) [file pone.0175407.s002.doc]

**S2 Fig 1**


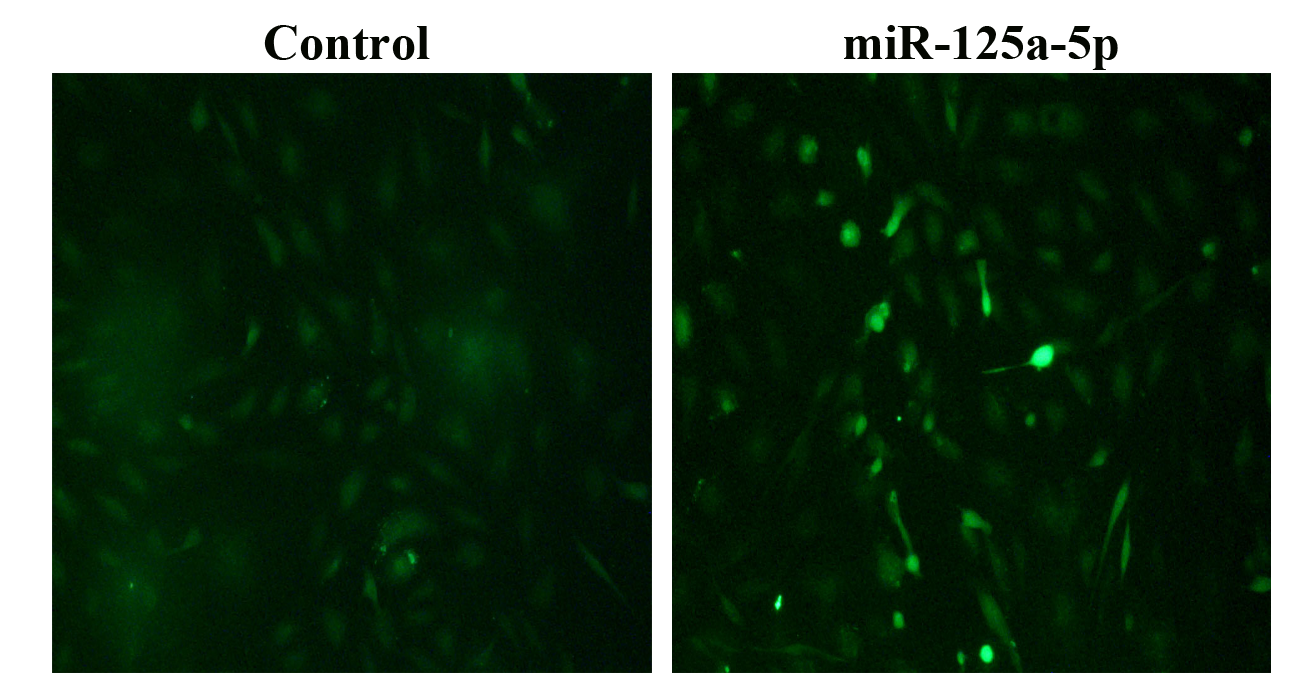
**A**

**B**

**C**

**S2 Fig 1. Transfection with miR-125a-5p expression vector or inhibitor in HUVECs.** A. HUVECs were transfected with miR-125a-5p-GFP expression or control vector, the transfection efficiency were observed by fluorescence microscope. The levels of miR-125a-5p were detected through real-time PCR while transfected with miR-125a-5p expression vector (B), or miR-125a-5p inhibitor (C) in HUVECs. ** p<0.05.
